# Donor Pooling as an Effective Method to Increase MSC EV Production Without Compromising Therapeutic Potential

**DOI:** 10.1002/jex2.70167

**Published:** 2026-07-13

**Authors:** Rebecca Davies, Claire Mennan, Anaïs Makos, Tian Lan, Charlotte Hulme, Mark Platt, Karina Wright, Oksana Kehoe

**Affiliations:** ^1^ Centre for Science and Technology in Medicine, School of Medicine Keele University Keele Staffordshire UK; ^2^ Robert Jones and Agnes Hunt Orthopaedic Hospital Oswestry Shropshire UK; ^3^ Centre for Science and Technology in Medicine, School of Life Sciences Keele University Keele Staffordshire UK; ^4^ Department of Chemistry, Centre for Analytical Science Loughborough University Loughborough UK

**Keywords:** donor pooling, extracellular vesicles, immunomodulation, inflammatory arthritis, mesenchymal stromal cells

## Abstract

Mesenchymal stromal cell extracellular vesicles (MSC EVs) hold great therapeutic potential. Their immunomodulatory abilities make them suitable candidates to treat autoimmune diseases, such as rheumatoid arthritis. However, MSC EV production must be reproducibly scaled to meet the demand of research and therapeutics. To achieve this, four human umbilical cord MSC (UC‐MSC) donors were pooled, a method known to generate a large cell source, that averages heterogeneous cell attributes. Upon generating MSC EV enrichments, donor pooling proved advantageous by increasing EV yield, both by increasing particle number and the presence of EV defining characteristics. Protein analysis suggests this could be due to an upregulation of protein transport mechanisms but requires further work to confirm. When applied to an inflammatory model of arthritis, pooled UC‐MSC EV enrichments surpassed their parental cells and single donor UC‐MSC EV enrichments, in alleviating arthritic pathophysiology, albeit the particle input was doubled since this was normalised by cell number. Therefore, we propose donor pooling as a simple and effective method to generate MSC EV enrichments, with potential to increase EV production without compromising therapeutic efficacy.

## 1 Introduction

Extracellular vesicles (EVs) are small, membrane bound particles that entrap biological cargo, such as protein and RNA, to govern paracrine signalling (Théry et al. 2009). Since the observation that mesenchymal stromal cells (MSCs) display immunomodulatory capabilities, independent of cell‐cell contact, it was proposed that delivery of paracrine signals alone could be of therapeutic benefit (Carlo‐Stella et al. 2002). MSC conditioned media exhibited such functional efficacy, with EVs being one of the mechanisms that contributed to this effect (Bruno et al. 2012; Kay et al. 2017). Today, MSC EVs are widely known to possess similar immunomodulatory abilities by inhibiting immune cell proliferation in a dose‐dependent manner and governing anti‐inflammatory effects (Chen et al. 2016; Del Fattore et al. 2015; Di Trapani et al. 2016; Ma et al. 2019 Umbilical cord MSCs (UC‐MSCs), specifically, are thought to have superior immunomodulatory abilities than other sources, but also benefit from higher proliferation capacity and lower immunogenicity, making them the preferred choice for cell therapies (Jin et al. 2013; Li et al. 2016).

Several animal models of autoimmunity have benefitted from the immunomodulatory abilities of MSCs, including graft versus host disease (GvHD), type I diabetes and inflammatory arthritis, with the latter displaying a reduction in joint swelling and improvement in histopathological outcomes (Cosenza et al. 2018; Fujii et al. 2018; Kay et al. 2021; Nojehdehi et al. 2018; Xu et al. 2021; Zhang et al. 2018). Similarly, MSC EVs have shown therapeutic promise in clinical trials of inflammatory immune disorders, such as chronic kidney disease and GvHD (Kordelas et al. 2014; Nassar et al. 2016). They are safe and well tolerated, leading to resolution of inflammation by encouraging a significant anti‐inflammatory plasma profile (increased transforming growth factor beta 1 (TGF‐β1), interleukin 10 (IL‐10), and decreased tumour necrosis factor alpha (TNF‐α)). Or an anti‐inflammatory peripheral blood mononuclear cell (PBMC) cytokine profile (decreased IL‐1β, TNF‐α and interferon gamma (IFN‐γ) positive PBMCs). This is in addition to the benefits of delivering an EV therapy over a cell one, including them being less biologically complex and malleable, easier to store, their improved safety profile and being small enough to easily permeate tissue (Caponnetto et al. 2017; Gelibter et al. 2022; Théry et al. 2009; Van Delen et al. 2024).

Despite this, several challenges must be addressed before MSC EVs become therapeutically viable. MSCs themselves are heterogeneous, which when used to produce a similarly diverse EV population makes it difficult to establish quantifiable parameters to define release criteria for an MSC EV product (Sacchetti et al. 2016; Wagner et al. 2006; Witwer et al. 2019). The EV field also suffers from issues in achieving sufficient yield, reproducibility and scalability which is essential to meet the demand of laboratory and clinical applications (Paolini et al. 2022; Phan et al. 2018). In consideration of this, we propose a pooled donor strategy of UC‐MSC EV production. Donor pooling is an existing strategy for MSC therapeutics where multiple single donors are combined. This averages the biological attributes of the resulting population, removing the need for donor selection and creates a large cell source, requiring fewer passages, to achieve a reproducible therapeutic product that is readily available as an ‘off‐the shelf’ therapy (Kannan and Gupta 2024; Ketterl et al. 2015; Kuçi et al. 2016; Mebarki et al. 2025; Samuelsson et al. 2009). There are also implications for manufacture, making the process more streamlined, consistent and cheaper.

Pooled bone marrow MSCs (BM‐MSCs) are comparable in terms of proliferation, survival and differentiation capacity in comparison to single donors (Kuçi et al. 2016; Widholz et al. 2019). For immunomodulatory abilities, pooled BM‐MSCs are equivalent to single donors in terms of modulating T‐cell activation and metabolic activity (Hejretová et al. 2020), and pooled BM, UC and adipose tissue (AT) sources have shown to be equivalent or even superior, to single donors in their ability to suppress allogeneic mononuclear cells (Kannan and Gupta 2024; Ketterl et al. 2015; Kuçi et al. 2016; Samuelsson et al. 2009). These results were highly reproducible across batches, emphasising the benefit of banking pooled donors. When trialled in the clinic for the treatment of GvHD, pooled BM‐MSCs were safe and well tolerated in children (Kurtzberg et al. 2020). For both adults and children, survival rates were much higher for severe GvHD patients when compared to other clinical trials using single donors (Bonig et al. 2019; Kuçi et al. 2016). This suggests that pooled MSCs can not only provide a large source of cells but may be more therapeutically beneficial.

**FIGURE 1:**
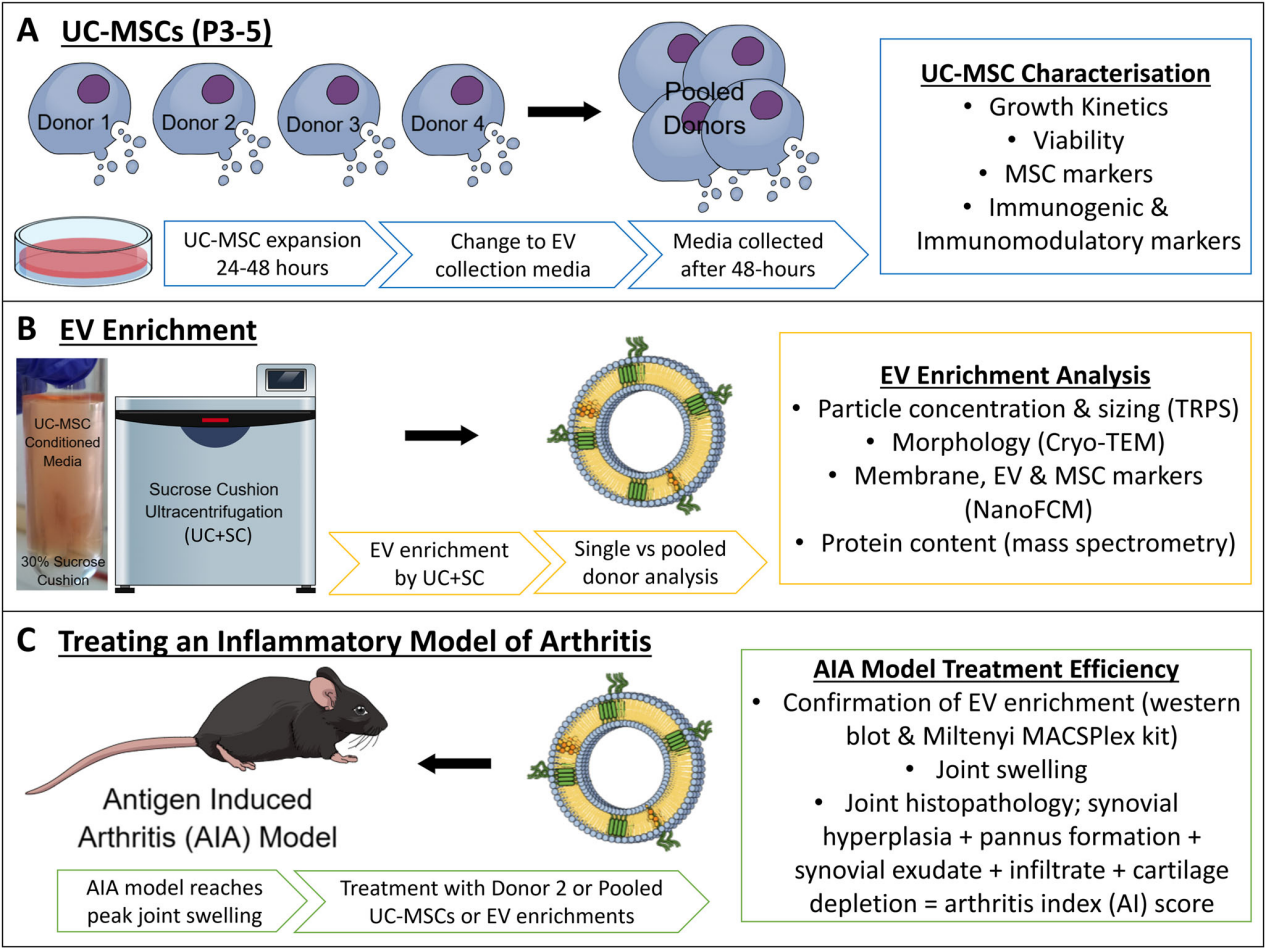
Experimental workflow. (a) A summary of how single (*n* = 4) and pooled donor UC‐MSCs were cultured and characterised, (b) EVs were enriched and analysed, and (c) how donor 2 and pooled donor UC‐MSCs and their EV enrichments, were used to treat an inflammatory model of arthritis and treatment efficiency was determined.

To date, no one has explored the implications of donor pooling on the resulting EV population, for MSCs or otherwise. Yet, pooling offers similar advantages for EVs as those described previously for cell therapies. They offer a large cell source to generate EVs which are likely to be more reproducible in terms of batch and donor variability. EVs may also be preferable over cells since the mismatch of human leukocyte antigen (HLA) donors is less likely to be an issue due to the low representation of a cell's surface markers on the available membrane (Galipeau et al. 2016; Théry et al. 2009). Hence, we compared the characteristics of UC‐MSC EV enrichments from single and pooled donor sources and tested their therapeutic potential using an in vivo inflammatory model of arthritis (Figure 1). This will contribute to the broader scientific knowledge of how donor pooling affects MSCs, the paracrine signalling occurring between them, and whether donor pooling may be a viable strategy to increase EV production for research and therapeutic use.

## 2 Materials and Methods

### 2.1 Isolation and Culture of UC‐MSCs

Human UC‐MSCs were processed as outlined in Mennan et al., 2013 and sourced from previous experiments (Mennan et al. 2019) in which they were expanded on tissue culture plastic before large‐scale expansion in the Quantum cell expansion system (Terumo, BCT, Millbrook, UK). Briefly, umbilical cords (*n* = 4) were sourced from the Robert Jones and Agnes Hunt Orthopaedic Hospital Maternity Unit following natural delivery, with full informed consent, REC reference 10/H10130/62 and collection of donor demographics (Table 1). Whole umbilical cord was weighed, minced, and digested with 1 mg/mL collagenase I (Sigma‐Aldrich, SCR103) for 1 h at 37°C. The tissue was removed and the resulting supernatant centrifuged at 80 x g for 10 min. The pellet was resuspended in MSC complete culture media, consisting of DMEM‐F12 (Fisher Scientific, 11330057), 10% foetal bovine serum (FBS) (Fisher Scientific, 10500064), and 1% penicillin‐streptomycin (Fisher Scientific, 15140122), and plated into tissue culture flasks. Upon confluency, UC‐MSCs were harvested and 5 × 10^6^ cells were seeded into the Quantum for the second expansion phase (P1). Further detail and full characterisation can be viewed for all UC‐MSC donor sources in Mennen et al., 2019. For this project, UC‐MSCs were later expanded on tissue culture plastic (P2‐5, Sanyo CO_2_ Incubator Model MCO 17A Serial 50704214, 5% CO_2_, 37°C), seeded at a density of 5 × 10^3^ UC‐MSCs per cm^2^. Cells were left to adhere for 24‐h, after which the 30 mL of media was replaced to remove non‐adherent cells. The UC‐MSCs did not require another media change until reaching 60% confluency (after 24–48 h, donor dependant) after which the media was exchanged in preparation for UC‐MSC EV enrichment (see Section 2.3). UC‐MSCs were evaluated for growth kinetics, cell viability and surface marker immuno‐profiles.

**TABLE 1:** Donor demographics (obtained at the time of gestational booking, roughly week 8–11), weight of tissue digested and cell harvest at P0 for UC‐MSC donors (Mennan et al. 2019).

UC‐MSC donor	Age of mother	BMI of mother	BMI classification	Gender of newborn	Tissue digested (g)	Cell harvest at P0 (x10^6^)
1	35	19.2	Healthy	Male	27	6.1
2	23	25.4	Overweight	Male	25	5.1
3	24	23	Healthy	Female	30	5.5
4	28	24.4	Healthy	Male	36	10.7

### 2.2 UC‐MSC Viability and Surface Marker Characterisation

Cell viability and surface marker characterisation was analysed using flow cytometry. Up to 1 × 10^6^ cells were resuspended in 100 µL of flow buffer (2% bovine serum albumin (Sigma‐Aldrich, A7030)) containing 5 µL F_C_ receptor block (BD Biosciences, 1018639) for 10 min at room temperature. This was made up to an appropriate volume of flow buffer and evenly distributed between flow cytometry tubes of 100 µL containing 3 × 10^4^ MSCs. Viability was assessed by adding 10 µL of 10 µg/mL propidium iodide (Sigma‐Aldrich, P4864) for 1 min before analysis. Surface markers were assessed by incubation with fluorochrome conjugated antibodies (BD Biosciences; see Table S1) for 30 min at 4°C in the dark. Samples were washed with phosphate‐buffered saline (PBS) and centrifuged at 350 x g for 8 min before resuspending in 250 µL flow buffer. Samples were analysed on the fluorescence‐activated cell sorting (FACS) Canto II cytometer (BD Biosciences) and the exported data analysed using FlowJo Software (version 10.7.1).

### 2.3 UC‐MSC EV Enrichment

Once cells reached ∼60% confluency, normally 24‐h later, although this could be up to 48‐h for slower donors at higher passage (i.e., donor 4 at passage 5) those being used for EV enrichment (P3‐5) were washed with PBS thrice before replacing the complete culture media with 20 mL of EV collection media, consisting of DMEM‐F12, 10% EV‐depleted FBS (ultracentrifuged FBS; 120,000 x g for 18 h) and 1% penicillin‐streptomycin, per T175. Generally, this would be conditioned by ∼3 × 10^6^ UC‐MSCs with >95% viability, reaching 80%–90% confluency upon harvest. Previous work in our laboratory has confirmed our UC‐FBS protocol successfully depletes FBS of 78.27% ± 4.85% of particles and 70.41% ± 6.68% of protein (Davies et al. 2023). It also provides further characterisation of the same single donor UC‐MSCs grown under UC‐FBS conditions, and how this compares to ultrafiltration and serum free methods. After 48‐h, the conditioned media was collected and centrifuged at 300 x g for 10 min, followed by a 2000 x g spin for 20 min at 4°C (Théry et al. 2006). The supernatant was then collected, with extra care being taken not to disturb the non‐visible pellet, ensuring it is covered in at least 500 µL of liquid. The collected supernatant was stored in ‐80°C for no longer than 1 month until EV enrichment.

Conditioned media for further processing was removed from the −80°C and thawed overnight (∼16 h) at 4°C. To bias small EVs <200 nm, conditioned media was syringe filtered through a 0.2 µm filter (Sarstedt, 501692591) before being loaded into open top thick‐wall polycarbonate tubes (Scientific Laboratory Supplies, S309140A). For EV enrichment, an improvised one‐step sucrose cushion ultracentrifugation method was used, which was established by Gupta et al. (Gupta et al. 2018). This methodology works based on the concept that the density of sucrose, 1.12–1.18 g/mL, overlaps with the density of small EVs, 1.15–1.19 g/mL, allowing them to be enriched within the cushion, and further separating protein contaminants of a higher density, 1.22 g/mL. The 30% sucrose cushion was made using 7.5 g sucrose (Merck, S0389), in which deuterium oxide (D_2_O, Merck, 151882) was added until the weight reached 30 g in total and placed on a roller for approximately 40 min. A single layer of sucrose cushion (2 mL) was layered on the bottom of the tube before spinning at 100,000 x g for 105 min at 4°C using a SW28 Ti Swinging‐Bucket Aluminium Rotor (Beckman Coulter, 342207). The sucrose cushion, enriched with EVs (2 mL), was then placed in polycarbonate bottles (Beckman Coulter, 355618), topped up with 200 nm filtered PBS (referred to as filtered PBS from hereon) and centrifuged at 100,000 x g for 70 min using a Type 70 Ti Fixed‐Angle Titanium Rotor (Beckman Coulter, 337922) to pellet EVs. The resulting supernatant was removed, and the pellet resuspended in either filtered PBS or serum‐free DMEM, depending on the downstream application. Samples that have undertaken this processing are referred to as ‘EV enrichments’ throughout. We use this terminology in consideration to the evidence presented in this paper than EVs are enriched after processing, but also to acknowledge the likely presence of non‐EV particulate. All EV enrichments were snap frozen in liquid nitrogen, stored at −80°C and their analysis completed within 1 month.

### 2.4 Pooling of UC‐MSCs and Harvest of the Conditioned Media for EV Enrichment

To generate a pooled donor source, UC‐MSCs were counted and combined in equal ratios (1:1:1:1 of the four single donors) at P1–2. This ‘pool’ was then expanded, as per section 2.1, on tissue culture plastic (P2–5). No analysis was conducted to determine the growth kinetics of individual donors throughout the culture of the pooled donor source. To generate UC‐MSC conditioned media to enrich EVs, the pooled donor was expanded to 60% confluency before exchange to the media used to enrich EVs, as per section 2.3.

### 2.5 Particle Quantification

Particle concentration and sizing was achieved by tunable resistive pulse sensing (TRPS) using Izon's qNano gold with a NP200 nanopore (Izon Science). As instructed by Izon, filtered PBS (or DMEM‐F12 in the case of EVs inputted into the antigen‐induced arthritis (AIA) model) was used to wet the lower and upper fluid cells and the nanopore stretch was calibrated using callipers. A stable amplitude of ∼130nA was achieved using 0.6 V and 47.56 mm stretch, in which 40 µL of sample was loaded. This was followed by running calibration particles (CP200, 1 × 10^9^–2 × 10^10^), under similar conditions, to calibrate samples for comparison. Data was recorded and analysed using the Izon Control Suite (version 3.4.2.48). Control samples were also run, including filtered PBS, serum free DMEM‐F12 and MSC culture media, processed as per Section 2.3, which were spiked with calibration beads at a known concentration, and used to verify that there was a minimal amount of particle contribution from fluids used to collect or resuspend EVs.

### 2.6 EV Lysis and Protein Quantification

EV enrichments were either resuspended in RIPA buffer (150 mM sodium chloride, 50 mM tris‐hydrochloride, 1% NP‐40, 0.5% sodium deoxycholate and 0.1% sodium dodecyl sulfate (SDS)) or 2% SDS depending on the downstream application. They were then kept on ice for 15 min, with vortexing and sonication occurring every 5 min. The total protein content of EV enrichments, or cells, resuspended in lysis buffer was determined using the bicinchoninic acid assay, as per the manufacturer's instructions (Fisher Scientific, 10741395). Generally, as a guideline to the input to downstream analysis, conditioned media generated from a T175 (20 mL EV collection media, ∼3 × 10^6^ UC‐MSCs) would be expected to yield a minimum of 3 µg protein, although the mean average was ∼5.28 µg. For example, 18 T175s were used, per sample, to generate an input of 60 µg into mass spectrometry analysis (Section 2.11).

### 2.7 Cryo‐Transmission Electron Microscopy

Cryo‐transmission electron microscopy (TEM) was outsourced to Dr Saskia Bakker at the University of Warwick Advanced Bioimaging Research Technology Platform as part of the Seedcorn Access Scheme (EP/V007688/1). Each sample (5 µL) was applied to a freshly glow‐discharged lacey carbon grid and plunge‐frozen using a Leica GP2 plunge‐freezer. Grids were imaged using a JEOL 2200 FS with a Gatan K2 camera. Scale bars were calibrated and attached using Fiji software (version 1.53c) (Schindelin et al. 2012).

### 2.8 Nano Flow Cytometry

Nano flow cytometry for analysis of single particles was outsourced to NanoFCM (Nottingham, UK) using their NanoAnalyzer. Briefly, samples were diluted to a particle concentration of 2 × 10^10^ particles/mL, of which 1 µL of the appropriate membrane dye and/or antibody at optimised dilutions were added to 9 µL of sample. Here, a combination of either the membrane dye MemGlow (Cytoskeleton, NG01‐10), or a cocktail of cluster of differentiation 9 (CD9), CD63 and CD81 antibodies (Abcam, see Table S1) were used to identify EVs, whilst CD73, CD90, CD105 or HLA‐DR antibodies (Abcam, see Table S1) identified surface markers. This was incubated for 30 min in the dark, room temperature, before the sample was run on the NanoAnalyzer. Data was acquired for 45 s before recording for a duration of 1 min, after which the samples were calibrated using standards of known size and subtraction of a blank containing the appropriate buffer.

### 2.9 Miltenyi MACSPlex Exosome Kit

The equivalent of 6 µg protein per sample was inputted into the MACSPlex Exosome kit (Miltenyi Biotec, 130‐122‐209), as per the manufacturer's recommendations. Briefly, EV enrichments (57 µL of donor 2, 45 µL of pooled donor) were diluted to 120 µL using the MACSPlex buffer before adding 15 µL MACSPlex Exosome Capture Beads and incubating overnight on an orbital shaker. Samples were washed by adding 500 µL of MACSPlex Buffer and centrifuging at 3000 x g for 5 min. A detection cocktail of CD9, CD63 and CD81 (5 µL each) added and incubated for 1 h at room temperature on an orbital shaker. Samples were washed twice again (as before), but with a 15‐min incubation for the second wash using the orbital shaker. Data was acquired on the FACS Canto II cytometer (BD Biosciences) and analysed using FlowJo Software (version 10.7.1).

### 2.10 Western Blot

Cell (10 µg) or EV lysate (18 µg) per sample was added 1:1 to 4x SDS Buffer (0.125 M Tris‐HCl, 4% SDS, 20% glycerol, 10% β‐mercaptoethanol and bromophenol blue) and heated at 90°C for 5 min with shaking. Samples were loaded onto a 4%–12% TGX strain‐free gel (Bio‐Rad, 4568084) and separated by electrophoresis before overnight transfer to a nitrocellulose membrane. Membranes were blocked for 2 h in 5% semi‐skimmed milk in Tris‐buffered saline with Tween‐20 (TBST) and incubated with primary antibodies (Fisher Scientific, Annexin α2/ANXA2: 1:2000, 03–4400, and Integrin β1/ITGB1: 1:4000, 14‐0299‐82) overnight at 4°C. After three 5‐min washes, the membrane was incubated with a secondary antibody (Fisher Scientific, Goat anti‐Mouse IgG (H+L), HRP: 1:4000, 62–6520) for 1 h at room temperature, before washing once more. SuperSignal West Femto Chemiluminescent Substrate (Fisher Scientific, 34094) was added to the membrane and imaged with ChemiDox Touch Imaging System using Image Lab software (v.6.0.1).

### 2.11 Sample Preparation for Mass Spectrometry

EV enrichments in PBS (60 µg per sample), lysed using a 1:1 ratio of 4% SDS, was inputted into the filter‐aided sample preparation (FASP) protocol. Three replicates representing an independent EV enrichment of conditioned media, from either single or pooled donor UC‐MSCs, was used. Samples were topped up to 400 µL using 8 M urea buffer and placed into Amicon ultra‐0.5 mL 10 kDa filters (Merck, UFC501024) before centrifuging. As with all centrifuge steps, samples were centrifuged at 8000 g for 20 min, room temperature, after which the filtrate was discarded. 400 µL of 8 M urea buffer was added to each sample, centrifuged and this step repeated once more before exchanging the urea buffer with 50 mM ammonium bicarbonate (ABC) by adding 400 µL and centrifuging three times. Reduction and alkylation were then conducted by adding 400 µL of 10 mM tris (2‐carboxyethyl) phosphine (TCEP, Sigma Aldrich, C0267) and 40 mM 2‐chloroacetamide (CAA, Sigma Aldrich, C4706) in ABC buffer and leaving for 30 min before centrifuging. The samples were washed using 400 µL 50 mM ABC buffer three times before adding 1.2 µg trypsin (Promega, V5111) resuspended in 200 µL ABC buffer per sample for on column protein digestion. Digestion occurred for 18‐h at 37°C before eluting the peptides once by centrifugation, and again by adding 400 µL of water and centrifuging for one final time. Peptides were concentrated using a SpeedVac at 42°C to reduce sample volume, to 30 µL, and stored at −20°C.

### 2.12 Mass Spectrometry

Mass spectrometry was outsourced to Dr Andrew Bottrill at the University of Warwick Proteomics Research Technology Platform as part of the Seedcorn Access Scheme (EP/V007688/1). Briefly, peptides were separated using reverse phase chromatography using two columns, an Acclaim PepMap μ‐precolumn cartridge 300 µm i.d. x 5 mm 5 µm 100 Å and an Acclaim PepMap RSLC 75 µm x 50 cm 2 µm 100 Å (Thermo Scientific) installed on an Ultimate 3000 RSLCnano system (Dionex). Eluted peptides were then converted to gas phase ions using electrospray ionization and analysed by a Thermo Orbitrap Fusion (Q‐OT‐qIT, Thermo Scientific). Tandem mass spectrometry was then performed by isolation using a quadrupole, High‐energy collision dissociation fragmentation and rapid scanning of the mass spectrometry analysis in the ion trap.

### 2.13 Mass Spectrometry Protein Identification

The resulting peptides were identified using the Uniprot human reference proteome, as well as the Uniprot bovine reference proteome to acknowledge the contribution of bovine proteins within EV enrichments. Scaffold proteome software (version 5.1.2) was then used to validate peptide identification by accepting those with a probability threshold of >95% and proteins with the same probability threshold and at least 2 identified peptides. This generated a list of proteins which were present in EV enrichments. These proteins were then inputted into the STRING database to output a visual network of protein‐protein interactions for each experimental condition (Szklarczyk et al. 2023).

### 2.14 Mass Spectrometry Data Analysis

Scaffold was also used for statistical analysis using an analysis of variance (ANOVA) test to determine which proteins were significantly upregulated between different experimental conditions, either single (*n* = 4) or pooled donors. To visualise the degree in which the proteome of experimental conditions varied from each other, normalised total spectra was used to calculate the mean of three replicates sources from different culture flasks and EV enrichments. This data was then transformed so that the mean was divided by the sum of means for each protein to give a percentage and visualised using a heat map generated using GraphPad Prism (version 9.1.0). To perform gene oncology (GO) analysis, the Panther classification system (version 17.0, accessed 04/08/23) was used to determine what proteins has been significantly enriched in the EV preparations using the human genome as a reference. The strength of the association between the identified proteins to the function pathways was determined using a Fisher's Exact test with Bonferroni correction.

### 2.15 Antigen‐Induced Arthritis (AIA) Model

For animal experiments, an AIA model was used, which is a T‐ cell driven, acute model of inflammatory arthritis that typically exhibits peak joint swelling at 24‐h post‐induction with clinical symptoms and histopathological signs that resemble rheumatoid arthritis. A murine AIA model was generated in 7–8‐ week old C57Bl/6 male mice, in accordance with Home‐Office approved project licence PPL number P0F90DE46 by Personal Licence Holder Dr Oksana Kehoe, as previously described (Jones et al. 2018; Kehoe et al. 2014). Mice were injected subcutaneously with an emulsion of 1 mg/mL methylated BSA (mBSA, Merck, A1009) in an equal volume of Freund's complete adjuvant (Merck, F5881) and intraperitoneally with 100 µL heat‐inactivated *Bordetella pertussis* toxin (Merck, P7208). This was repeated a week later. AIA was then induced, 21 days after the first injection (experimental day 0), by injecting mice intraarticularly with 10 mg/mL mBSA in PBS in the right knee joint, with the same volume of PBS being injected in the left knee to act as a control.

After 24‐h post arthritis induction (experimental day 1), when the right arthritic knee joint reached peak swelling, mice were injected intraarticularly, through the patellar ligament into the right joint, with 15 µL of control (serum‐free DMEM‐F12, *n* = 13), or one of the experimental treatments: either 5 × 10^5^ donor 2 UC‐MSCs (*n* = 9), 5 × 10^5^ pooled UC‐MSCs (*n* = 4), or an equivalent amount of EV enrichments generated from the same number of cells resuspended in serum‐free DMEM‐F12 (*n* = 4 for donor 2 and pooled EVs, or *n* = 6 for joint measurements of pooled EVs only). Here, stretching of the hind‐leg facilitated the intraarticular injection. For this experiment, EV enrichments were generated by 48‐h conditioning of UC‐MSCs in serum‐free culture media and enriched using differential ultracentrifugation with sucrose cushion under sterile conditions.

### 2.16 Joint Swelling and Histological Assessment of the AIA Model

The diameter of knee joints was measured every 24‐h using a digital micrometre (Kroeplin GmbH) with swelling being calculated by subtracting the joint diameter of the left control knee from the right arthritic knee. At the experimental end point, day 3 post‐arthritis induction, mice were sacrificed. Their joints were fixed in 10% neutral buffered formal saline, decalcified in formic acid and paraffin embedded to generate mid‐sagittal sections of 5 µm thickness. Sections were stained with haematoxylin and eosin (H&E) or toluidine blue, as described previously (Kay et al. 2017). H&E sections were used to score synovial hyperplasia and pannus formation (0–3, normal to over three layers thick with overgrowth and cartilage loss of the joint), synovial exudate (0–3, normal to substantial leukocyte number and large fibrin deposits) and infiltrate (0–5, normal to ablation of synovial adipose tissue by the large presence of inflammatory infiltrate). Toluidine blue sections were used to score cartilage depletion (0–3, normal to destruction of articular cartilage with significant erosion of the bone). A sum of all four parameters was used to calculate Arthritis Index (AI) (Jones et al. 2018).

### 2.17 Statistical Analysis

GraphPad Prism was used for data analysis and graphical presentation (version 9.1.0). Details of the statistical tests used will be stated throughout but, generally, data sets were tested for normality using a Shapiro‐Wilk test. Depending on the result, and the data to be analysed, further testing was conducted to determine level of significance. Typically, for the comparison of two experimental conditions, a *T*‐test was used for normally distributed data (parametric), whilst a Mann–Whitney test was used for data that was not (non‐parametric). If more than two comparisons were to be made then ANOVA (parametric) or Krustal–Wallis tests (non‐parametric) were conducted, typically with follow up testing to make multiple comparisons within the data set. A *p*‐value < 0.05 was considered significant and indicated using asterisks; *p < 0.05, **p < 0.01, ***p < 0.001 and ****p < 0.0001.

## 3 Results

### 3.1 Pooled Donor UC‐MSCs Display Similar Characteristics to Single Donors

**FIGURE 2:**
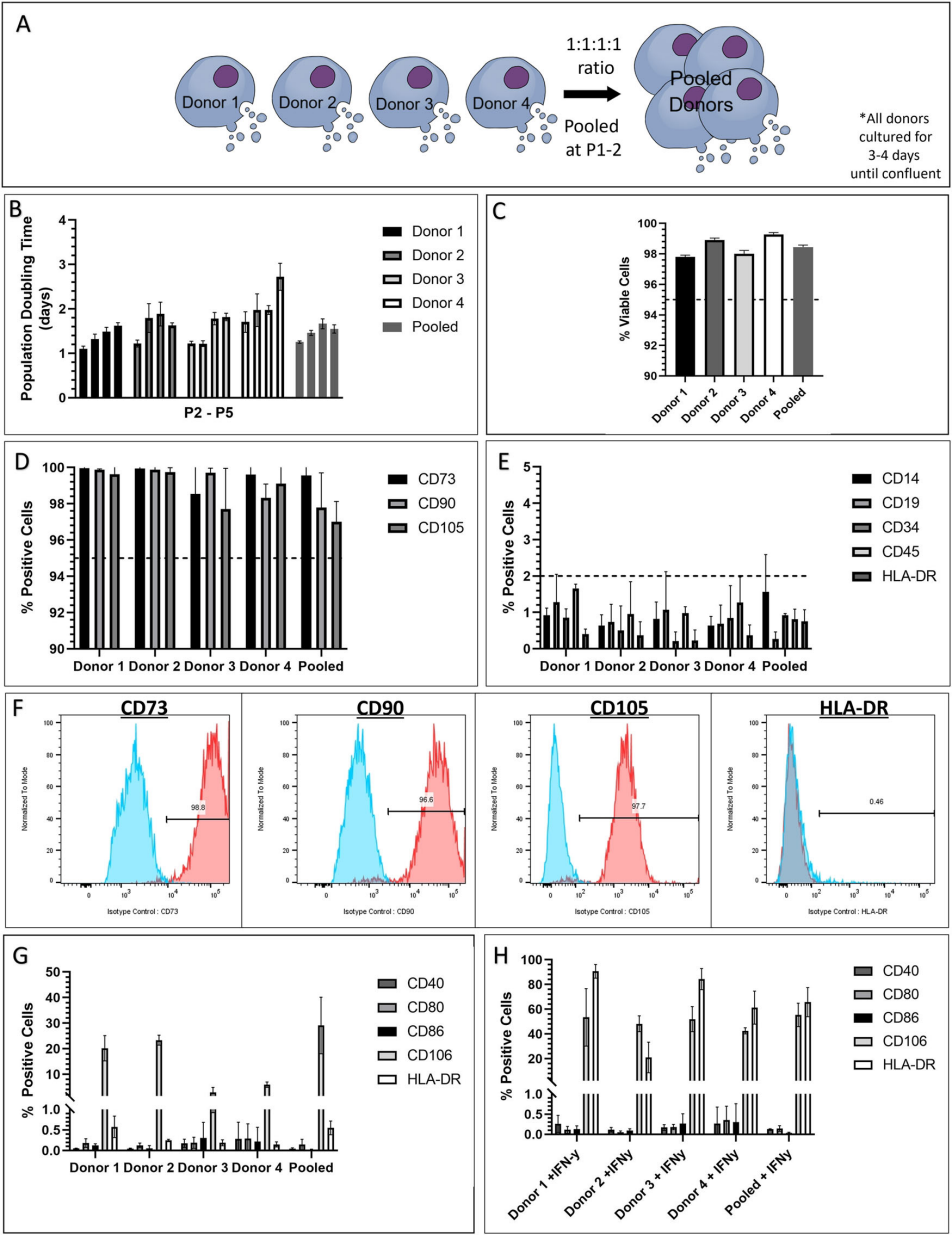
Characterisation of UC‐MSCs. (a) A visualisation of the four single donors used to generate the pooled donor source. All donor sources were evaluated for (b) P2–5 population doubling time, *n* = 5, (c) cell viability, *n* = 5, (d) MSC positive surface markers, *n* = 3, (e) MSC negative surface markers, *n* = 3, (f) representative histograms of CD73, CD90, CD105 and HLA‐DR showing marker positivity (red) and an isotype control (blue), (g) immunogenic and immunomodulatory markers without IFN‐γ activation, *n* = 3, (h) or with IFN‐γ activation, *n* = 3. Data presented as mean ± SD with replicates representing cells from independent culture flasks.

Cell characteristics were assessed to explore how a pooled donor strategy affected UC‐MSCs and to give context to the resulting EV enrichment source (Figure 2a). Donor pooling ‘averaged’ the population doubling time seen in individual UC‐MSC donors, being able to compensate for the slower proliferation of donor 4 at passage 5 (Figure 2b). Yet, pooled UC‐MSCs retained a high cell viability of >95% and maintained the presence of typical MSC surface markers, as defined by the International Society of Cell and Gene Therapy (ISCT) (Figure 2c–f) (Dominici et al. 2006).

Several immunogenic and immunomodulatory markers were also assessed. Immunogenic markers included CD40, CD80 and CD86, all of which are present on antigen presenting cells and promote T‐cell activation (Hancock et al. 1996). Regardless of whether UC‐MSCs were IFN‐ƴ activated or not, both single and pooled MSCs showed <1% positivity of these markers (Figure 2g, h). HLA‐DR was detected in <1% of non‐activated MSCs only and was upregulated upon IFN‐ƴ activation with the pooled donor reflecting an average of the single donors (mean, 65.70% ± 11.75% for pooled donor, 64.33 ± 31.51 for the average of four single donors). The immunomodulatory marker, CD106, was also assessed (Yang et al. 2013). Large variation was identified in single donors (range, 2.88% ± 1.97% to 23.33% ± 1.96%), while the pooled donor displayed a high CD106 positivity of 29.07% ± 11.04%. This variability disappeared once MSCs were IFN‐ƴ activated (mean, 55.43% ± 9.47% for pooled donor, 49.04% ± 4.86% for the average of all four single donors).

### 3.2 Pooled Donor UC‐MSC Conditioned Media Enriched More EVs in Comparison to Single Donors

**FIGURE 3:**
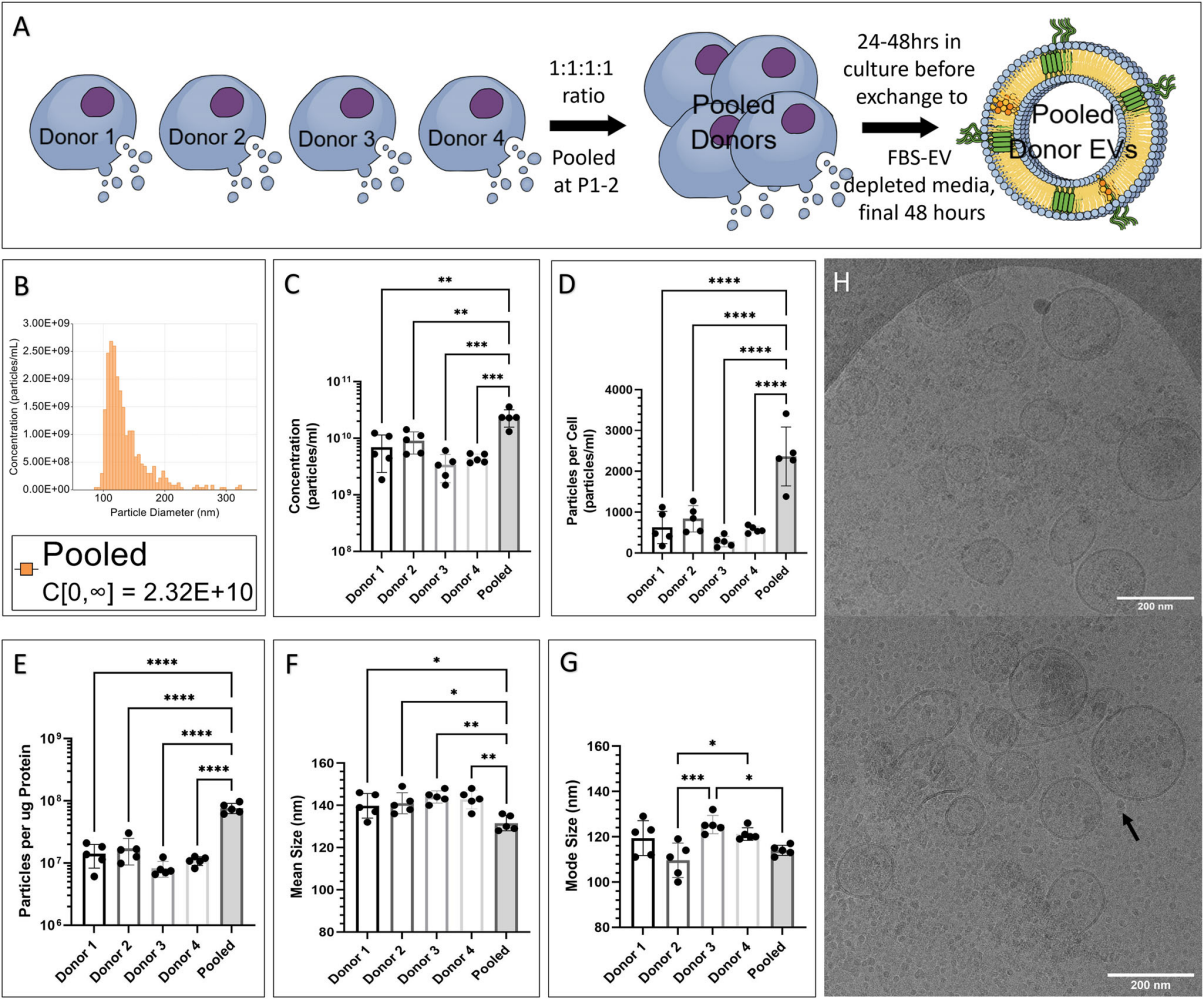
Particle characterisation of UC‐MSC EV enrichments. (a) A visualisation of the four single donors and pooled donor strategy used to generate EV enrichments. All sources were evaluated for (b, c) particle concentration, with a representative histogram of a pooled donor sample, (d) particles per cell, (e) particles per µg protein, (f) mean particle size, (g) mode particle size, (h) morphology by cryo‐electron microscopy with an arrow indicating the small, <20 nm, electron dense particles seen only in pooled EV enriched samples only. Data presented as mean ± SD with replicates, *n* = 5, representing EV enrichments from independent culture flasks. Statistical testing was conducted using a Shapiro–Wilk normality test and a one‐way ANOVA with Tukey correction for multiple comparisons with results indicated by *p < 0.05, **p < 0.01, ***p < 0.001 and ****p < 0.0001.

The media collected from UC‐MSCs was subjected to EV enrichment using differential ultracentrifugation with a 30% sucrose cushion (Figure 3a). Upon evaluation of particle concentration by TRPS, donor 2 had the most particles, 9.06 × 10^9^ ± 3.83 × 10^9^, whilst donor 3 had the least, 3.38 × 10^9^ ± 1.75 × 10^9^, although there was no significant difference between single donors (Figure 3b, c). This was similarly true for particle: protein ratios, mean particle size and mode particle size (Figure 3e–g), except for donor 2 having a lower mode size in comparison to donors 3 and 4 (Figure 3 g, *p* < 0.001, *p* < 0.05 respectively). Pooled donor samples contained significantly more particles, 2.36 × 10^10^ ± 8.02 × 10^9^, than all four single donors (*p* < 0.01), that which significantly increased particle: protein ratios (*p* < 0.001) and decreased mean particle size (*p* < 0.05). This was not a consequence of cell number, as normalising for this similarly displays these results, with more particles being significantly enriched per cell (Figure 3d, *p* < 0.0001).

Upon assessment of particle morphology, in general, many particles were membranous with a typical double lipid bilayer structure, of which the majority were single vesicles, with other morphologies present (double vesicles, multivesicular bodies, actin filled tubules, coated vesicles and large, pleomorphic vesicles) (Figure 3h) (Emelyanov et al. 2020; Höög and Lötvall 2015; Yuana et al. 2013). Yet non‐vesicular extracellular particles were also identified and were suspected to be common EV co‐isolates (lipoproteins and protein aggregates). Regardless, most particles being EV‐like, with a background of non‐EV particulate, was true for all samples. Any differences in representative images were likely to be random, based on the field of view, as expressed by a third party (Dr Saskia Bakker, University of Warwick) who was both blinded and unbiased to the nature of the work. The only exception to this was the high presence of small (<20 µm) electron dense particles in pooled donor samples (arrow, Figure 3h).

**FIGURE 4:**
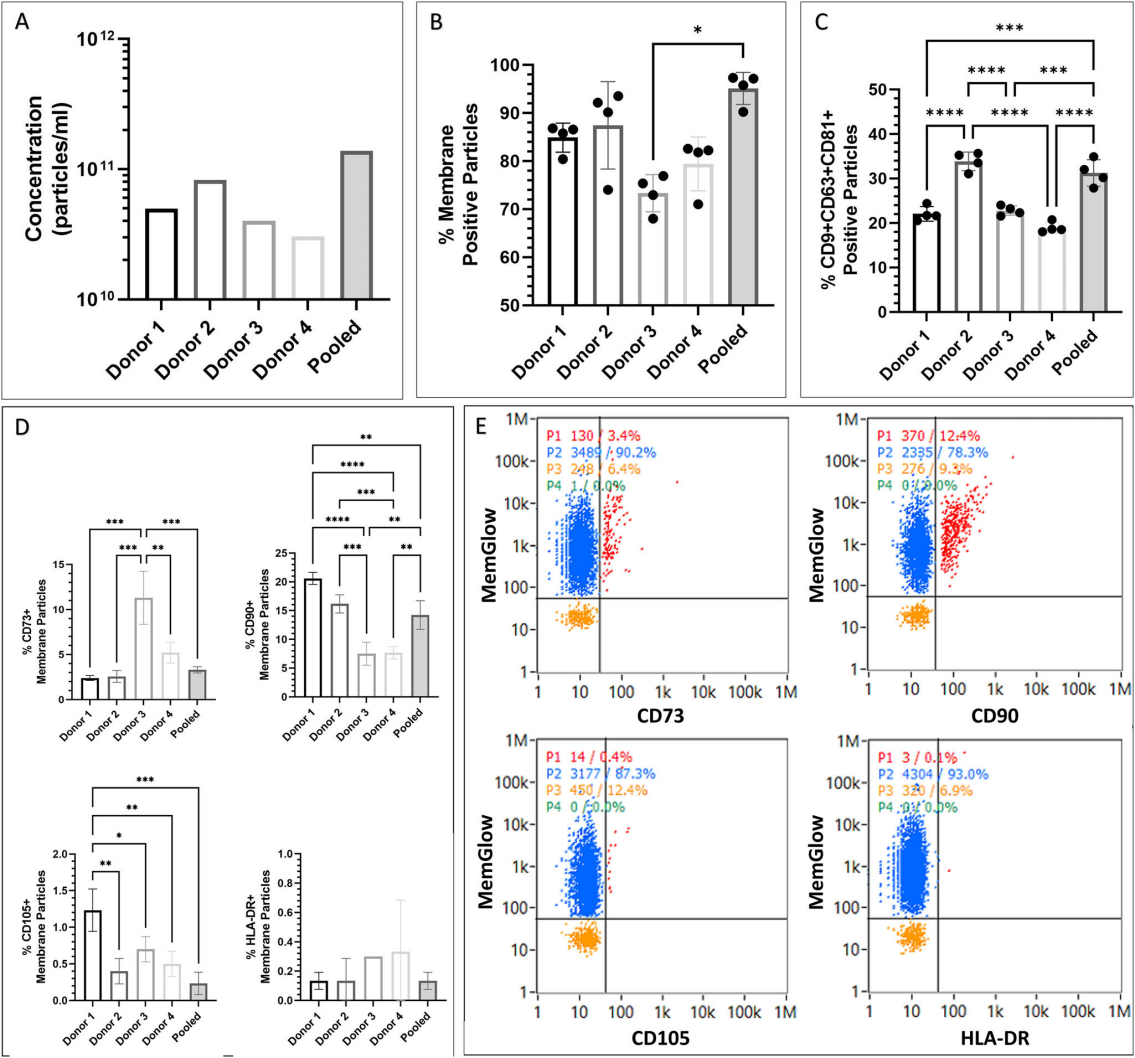
Surface marker characterisation of UC‐MSC EV enrichments. All donor sources, single and pooled, were evaluated for (a) particle concentration determined by the NanoAnalyzer, (b) percentage of membrane positive particles, (c) percentage of CD9, CD63 and CD81 positive markers (d) percentage of CD73, CD90, CD105 and HLA‐DR positive particles (e) with representative plots. Data presented as mean ± SD with technical replicates, *n* = 4. Statistical testing was conducted using a Shapiro–Wilk normality test, (b) a Kruskal–Wallis test with Dunn correction for multiple comparisons, and (c, d) a one‐way ANOVA with Tukey correction for multiple comparisons with results indicated by *p < 0.05, **p < 0.01, ***p < 0.001 and ****p < 0.0001.

Supplementary to this visualisation, nano flow cytometry was used to determine the proportion of membrane positive particles <200 nm, or those containing common EV markers, CD9, CD63 or CD81 (Figure 4a–e). Here, the pooled donor source was confirmed to contain the highest number of particles (*data not shown*, 1.39 × 10^11^ particles/mL in pooled donor, range of 3.05 × 10^10^ (donor 4) to 8.31 × 10^10^ (donor 2) particles/mL for single donors). The pooled donor sample also contained the highest proportion of membrane positive events (Figure 4b, mean, 95.12% ± 3.33% for pooled donor, range of 73.31% ± 3.88% (donor 3) to 87.45% ± 9.07% (donor 2) for single donors). This trend was also displayed for EV markers, although at a much lower frequency (Figure 4c, mean, 31.25% ± 2.97% for pooled donor, range of 18.96% ± 1.21% (donor 4) to 33.86.45% ± 2.08% (donor 2) for single donors).

Further to typical EV characterisation, it has been hypothesised that due to the high representation of MSC markers, CD73, CD90 and CD105, in cells, such antigens could be used to define MSC EVs (Witwer et al. 2019). To explore this further, the proportion of double positive particles, that are both membrane‐bound and contain positive MSC markers, or HLA‐DR, was investigated (Figure 4d, e). HLA‐DR was selected as the chosen negative marker in the interest of exploring a marker that plays a role in immune modulation (Galipeau et al. 2016). Generally, MSC positive markers were not highly abundant in MSC EVs and had wide variation between single donors, with CD90 being most present (range, 7.5% ± 1.99% to 20.6% ± 1.05%) and CD105 being least present (range, 0.40% ± 0.17%–1.23% ± 0.29%). The pooled donor source seemed to represent an average of these values (for example, CD90 was expressed in 14.23% ± 2.49% of the pooled donor) but HLA‐DR had little expression (<1%) in any sample.

### 3.3 Pooled Donor UC‐MSC EV Enrichments Promoted a Positive Regulation of Transport Mechanisms

**FIGURE 5:**
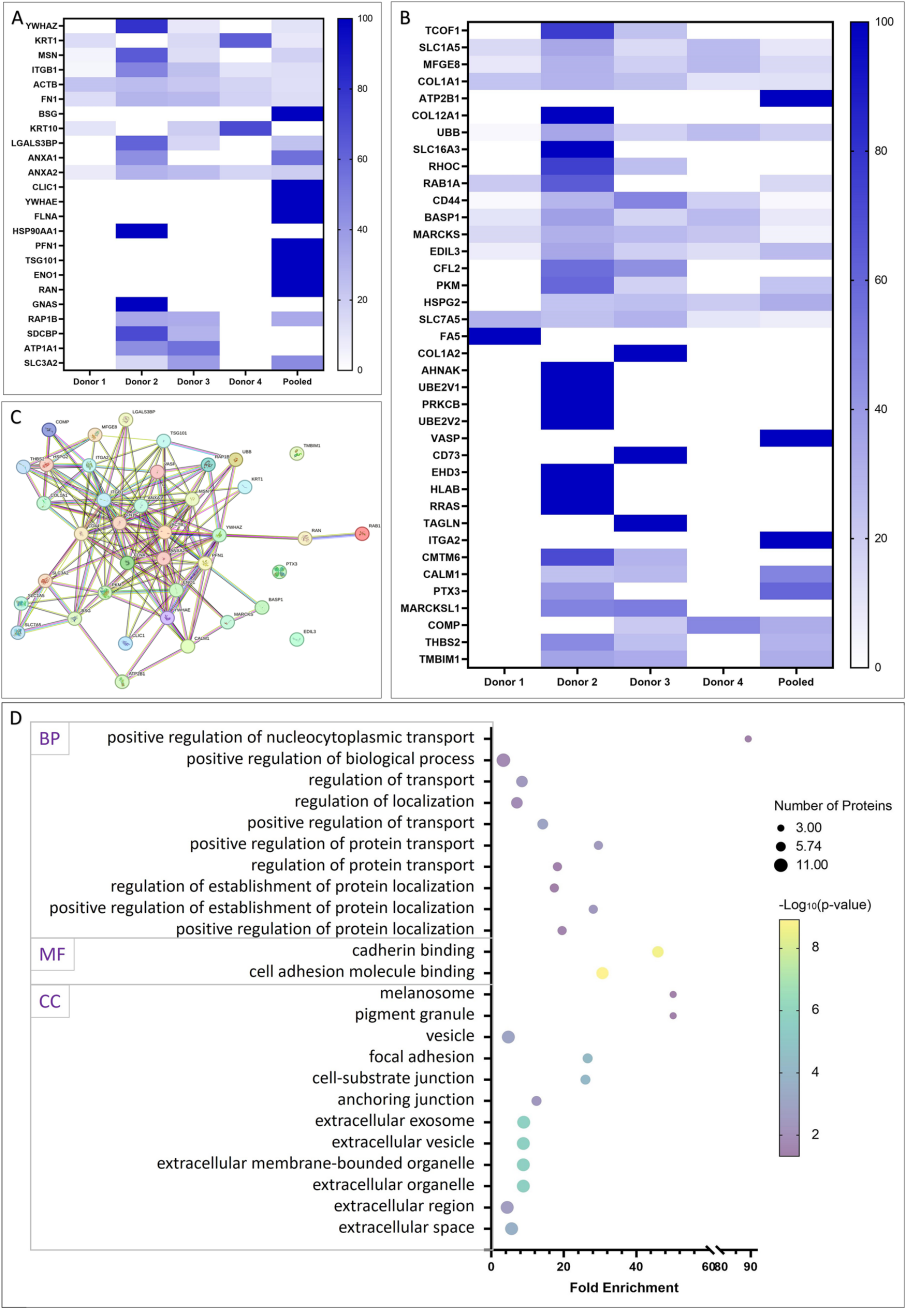
Proteomics of UC‐MSC EV enrichments. All donor sources, single and pooled, were submitted for proteomic analysis to identify (a) the proportion in which common vesicular proteins, as defined by Vesiclepedia, are present in each donor source, (b) the proportion in which other proteins are present in each donor source. The pooled donor source showed the presence of many proteins (c) represented in an interaction map, (d) which were associated with many significantly enriched terms identified in GO analysis. Data presented (a, b) with colour scales that represent the mean normalised total spectra of replicates (*n* = 3), from independent culture flasks, which has been transformed into a percentage by division of the sum of means for each protein identified, and (c) a dot in which colour represents the log transformed significance of the enrichment and size represents the increasing number of proteins associated with each term found in the analysis.

A wider view of the protein content of the EV enriched preparations was revealed using mass spectrometry (Figure 5a–d). All donor sources displayed several EV associated proteins, as defined by Vesiclepedia (Kalra et al. 2012; Pathan et al. 2019). This was confirmed by GO analysis with the most significant terms identified in cellular components being ‘extracellular vesicle’ or similar variations, along with other EV‐associated biological or molecular processes (Figure S2). Of note, in support to the therapeutic benefit of MSC EV enrichments, was the >10‐fold enrichment of the term ‘wound healing’.

Specifically, in pooled donor UC‐MSC EV enrichments, 39 human proteins were detected overall (Figure 5c). Despite a large overlap of protein clusters between the single and pooled donors, there were many EV‐associated proteins not detected in single donor sources (Figure S1, Figure 5a). Of the proteins detected, two were significantly upregulated, CD147 and plasma membrane calcium‐transporting ATPase 1 (ATP2B1) (Figure 5c, *p* < 0.05). GO terms exclusive to pooled donor EV enrichments included several associated with a positive regulation of transport mechanisms and movement of small molecules, ions and proteins, within or between cells (Figure 5d).

### 3.4 Pooled Donor UC‐MSC EV Enrichments Effectively Reduced Joint Swelling and Histopathological Signs in a Mouse Model of Inflammatory Arthritis

We have previously demonstrated the efficacy of MSCs, MSC conditioned media and pro‐inflammatory primed EV enrichments isolated from human bone marrow aspirates in ameliorating swelling and improving histopathological outcomes in an AIA model (Kay et al. 2017; Kay et al. 2021; Kehoe et al. 2014). In this study, we wanted to determine the ability of a pooled donor source of UC‐MSCs, and their derived EV enrichments, in treating this AIA model. To compare with the pooled donor source, donor 2 was selected as a single donor representative. This was based on donor 2's ability to suppress activated PBMCs and the highest presence of immunomodulatory markers, CD106 and indoleamine 2,3‐dioxygenase (IDO), post‐inflammatory licencing (Figure 2h, (Davies et al. 2023)), both of which are associated with promoting immunosuppression by altering cytokines, T‐cell subsets, and immune cell metabolism (Guan et al. 2018; Krampera et al. 2013; Meisel et al. 2004Ren et al. 2010; Yang et al. 2013).

**FIGURE 6:**
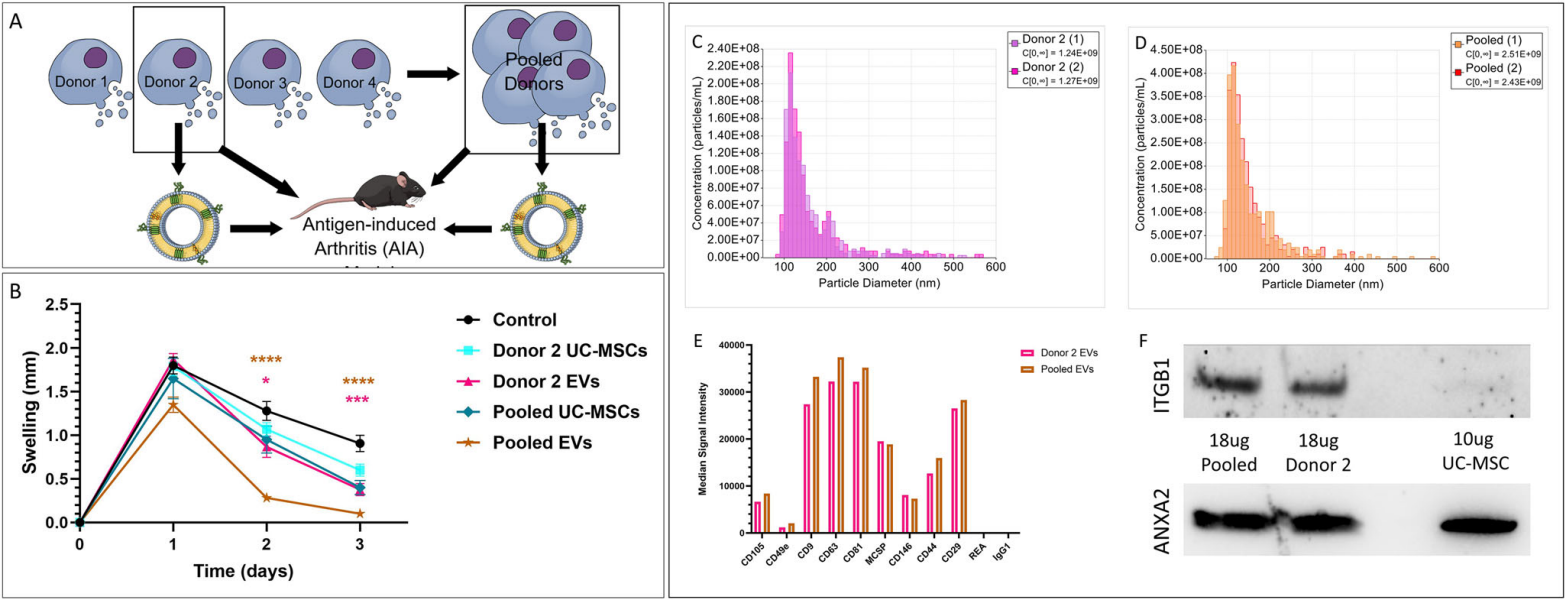
Treatment of an inflammatory model of arthritis with UC‐MSC EV enrichments. (a) A visualisation of the experiment in which either a control (*n* = 13), donor 2 (*n* = 9), or pooled UC‐MSCs (*n* = 4), or their derived EVs (donor 2 *n* = 9, pooled *n* = 6), were used to treat an antigen‐induced arthritis (AIA) model. For each treatment group, (b) the model was evaluated for joint swelling. For the UC‐MSC EVs enrichments used, (c) donor 2 and (d) pooled sources were evaluated for particle concentration, (e) surface markers, and (f) ITGB1 and ANXA2 by western blot. Data presented as mean ± standard error of the mean. Statistical testing was conducted using a Shapiro‐Wilk normality test and a one‐way ANOVA with Tukey correction for multiple comparisons with results indicated by *p < 0.05, **p < 0.01, ***p < 0.001 and ****p < 0.0001.

Donor 2, and pooled UC‐MSCs, were incubated with serum free DMEM for 48‐h to generate EV enrichments that could be used to treat an AIA model (Figure 6a–f). To provide an equivalent to an input of 5 × 10^5^ cells (Kay et al. 2017; Kehoe et al. 2014), EV enrichments were resuspended in serum free DMEM so that 15 µL was equivalent to EVs from the same number of cells. This generated a concentration of ∼1.25 × 10^9^ particles/mL for donor 2 and ∼2.47 × 10^9^ particles/mL for pooled UC‐MSCs (Figure 6c, d). Therefore, ∼1.88 × 10^7^ particles from donor 2 and ∼3.71 × 10^7^ particles from pooled donors was delivered to each mouse, values considered to be optimal for EV delivery in inflammatory arthritis (Wei et al. 2024). These particles similarly showed the presence of common EV markers CD9, CD63 and CD81 and other MSC markers, as well as the presence of ANXA2 and ITGB1 which was confirmed by western blot (Figure 6e, f).

**FIGURE 7:**
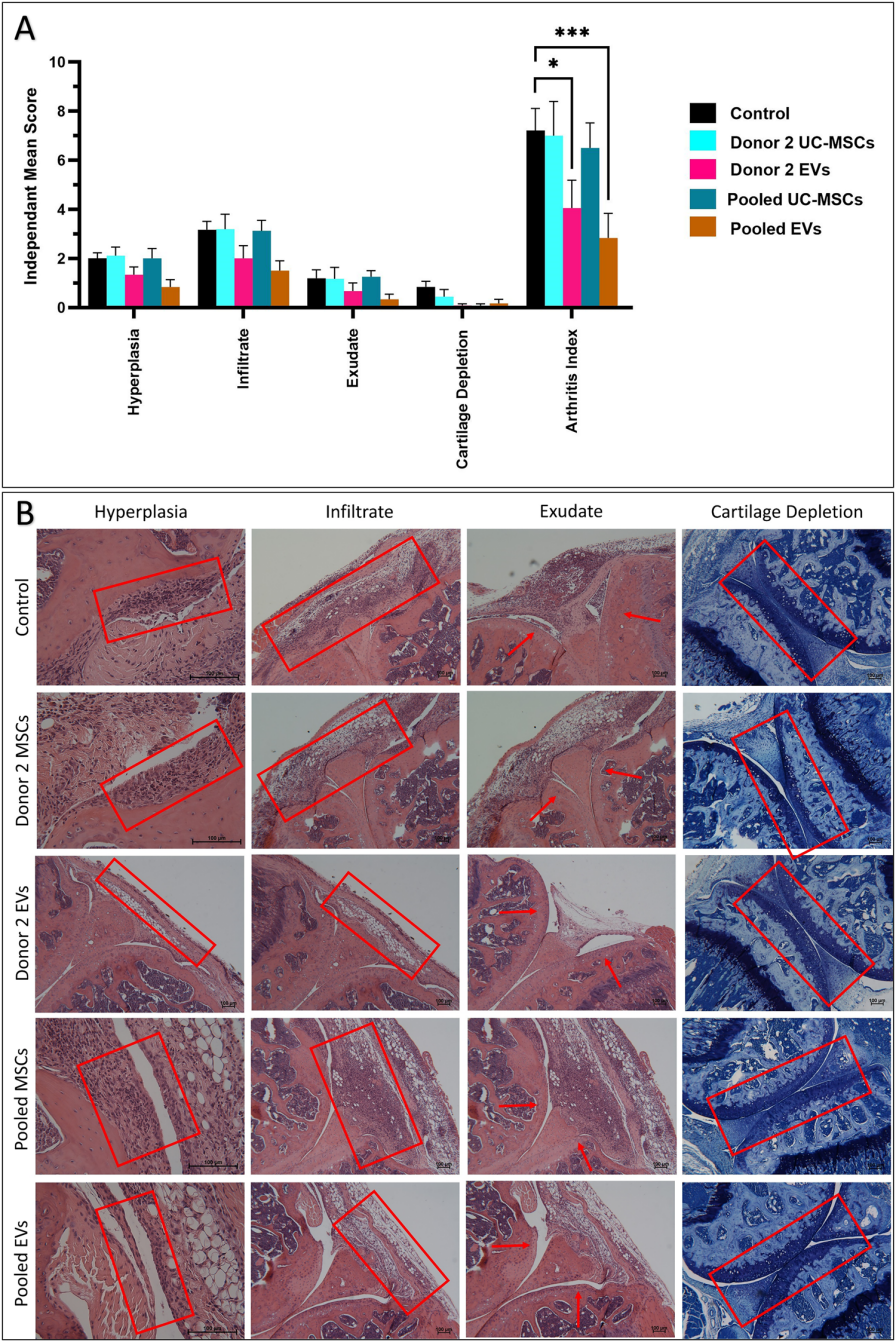
Histopathology of murine joints in an inflammatory model of arthritis treated with UC‐MSC EV enrichments. (a) Quantitative scores of hyperplasia (where the lining of the joint, or synovium, should be one cell thick, but increases due to proliferation in inflammatory conditions, cells are stained dark purple), infiltrate (of immune cells, stained dark purple), exudate (fluid and cells leaked from blood vessel in inflammation, depicted by dark purple in the white joint spaces), cartilage depletion (cartilage is stained in dark blue, but becomes pale when depleted), and AI, which is a sum of all four scores, with (b) representative images of each experimental treatment. Data presented as mean ± SD. Statistical testing was conducted using a Shapiro‐Wilk normality test and a one‐way ANOVA with Tukey correction for multiple comparisons with results indicated by *p < 0.05, **p < 0.01, ***p < 0.001 and ****p < 0.0001.

After 24‐h, the peak of joint swelling, an injection of 15 μ was administered into the joint which consisted of serum free DMEM‐F12 (control), 5 × 10^5^ donor 2 UC‐MSCs, 5 × 10^5^ pooled UC‐MSCs, or the equivalent particle number of EV enrichments. At day two and three, joint swelling, a measure of inflammation, was significantly reduced in mice treated with EV enrichments only, in comparison to the control (Figure 6b, *p* < 0.05 for donor 2 EVs, day 2, *p* < 0.001, day 3, and *p* < 0.0001 for pooled EVs on both days). At day 3, joints sections were assessed for histopathological symptoms of AIA including immune cell infiltration into the synovium, hyperplasia of the synovial membrane, extravasation of leukocytes into the synovial joint cavity and cartilage depletion. Whilst UC‐MSCs showed a tendency to reduce cartilage depletion compared to a serum‐free DMEM control, this was not statistically significant (Figure 7a, b). In comparison, AI, a sum of all four indicators to disease histopathology, showed significant reductions in mice treated with donor 2 (4.06 ± 1.13) and pooled donor MSC EV enrichments (2.83 ± 1.01) compared to serum‐free DMEM controls (7.21 ± 0.94) (Figure 7a). Here, a lower score represents an improvement in disease severity (Jones et al. 2018).

## 4 Discussion

Donor pooling offers many advantages to the world of cell therapy, being able to create an abundant MSC source, ‘average’ their heterogeneous nature, thereby mitigating donor variability and potentially enhance their immunomodulatory properties (Hejretová et al. 2020; Kannan and Gupta 2024; Ketterl et al. 2015; Kuçi et al. 2016; Kurtzberg et al. 2020; Mebarki et al. 2025; Samuelsson et al. 2009; Widholz et al. 2019. We have provided further evidence to support this, showing that MSC phenotype does not change upon pooling and biological attributes that tend to vary amongst this cell type, such as growth kinetics, are ‘averaged’. Donor pooling was also found to have no effect on MSC viability and did not promote an upregulation of markers associated with T‐cell activation and immunogenicity (Hancock et al. 1996; Mennan et al. 2016). This was true, even upon the introduction of inflammatory stimuli, recommended by ISCT to indicate MSC potency (Galipeau et al. 2016). In the case of CD106, we have previously shown that this surface marker is present in low abundance on UC‐MSCs, but increases after pro‐inflammatory priming (Hyland et al. 2023). Yet, CD106, in the absence of an inflammatory stimuli, was variable in this study and donor pooling did not seem to ‘average’ its abundance, showing equivalent or possibly higher presence in pooled MSCs, although it is uncertain to say which due to the high standard deviation seen amongst replicates.

These finding are, perhaps, unsurprising since donor pooling has proven promising enough to establish MSC banks of high viability upon resurrection and is at the stage of being tested in stage III clinical trials (Bonig et al. 2019; Kuçi et al. 2016; Kurtzberg et al. 2020). Furthermore, MSCs have been widely reported not to express such T‐cell activation markers, and another study has confirmed this after donor pooling (Lu et al. 2005; Mebarki et al. 2025). The level of CD106 in pooled donors could even be indicative of a superior immunomodulatory profile since this marker enables T‐cell adhesion, represents an MSC population that prevents IFN‐ƴ and TNF‐α secretion in PBMCs and promotes immunosuppressive T‐helper subsets (Ren et al. 2010; Yang et al. 2013). Although such a relationship was not established in another donor pooling study analysing CD106 (Mebarki et al. 2025). In this study, it was confirmed that the benefit of ‘averaging’ their immunomodulatory capabilities was retained. Despite the progress and promise of pooled MSCs, the complexity of MSC potency and mechanism means no one has yet explored how pooling could change this.

In the interest of this largely neglected, but fundamental research question, the primary focus of this work was to determine the effect donor pooling had on the resulting MSC EV enrichments. First, MSC EVs were established to be successfully enriched due to the bias towards small particles, <200 nm, abundance of several EV markers, and typical EV morphology (Emelyanov et al. 2020; Höög and Lötvall 2015; Welsh et al. 2024; Yuana et al. 2013). Non‐vesicular extracellular particulate was present, which was expected given the acknowledgement that no EV methodology to date can truly isolate them without impurities (Théry et al. 2018; Tian et al. 2020; Welsh et al. 2024). However, the degree of contamination from EV‐depleted collection media should, theoretically, be the same in all samples, thereby retaining the validity of these experiments. Thus, in comparison to single donor sources, donor pooling was capable of increasing MSC EV yields, both by establishing a larger cell source in which to generate EVs from and how, in this specific pool, there was an increase in EV production per cell. This was based on the increase in particle yields which made it easier to achieve a higher ‘purity’ and enhance the enrichment of commonly associated EV tetraspanins (CD9, CD63 and CD81). In fact, primary MSCs, in this and other studies, seemingly produce a range of 100–1000 particles per cell, of which pooled donors are easily able to achieve twice the upper range of this value (Haraszti et al. 2018; Kim et al. 2022; Tang et al. 2021).

EVs are considered mediators of cell‐cell communication (Bazzoni et al. 2020), so it is possible that there is an underlying recognition of non‐self which could illicit such a reaction. Yet, this does not necessarily mean the consequences for therapeutics would be negative. Foetal MSCs are known to be more tolerant to donor mismatching, for example. Such a reaction may convey therapeutic benefit when considering the promise of research dedicated to immunomodulatory superiority following inflammatory priming (Chang et al. 2006; Kim et al. 2018; Prasanna et al. 2010). However, extensive work would be needed to confirm the optimal donor pool strategy and ensure the safety of a pooled donor source, although the finding that an allogeneic source of EVs has a comparative safety profile to autologous EVs is promising (Van Delen et al. 2024). Alternatively, this could present a further advantage for delivering an EV therapy over a cell one. Pooled donor cell sources, particularly, are at high risk of alloreactivity and disease transmission, meaning pooled donor EV enrichments may provide a safer alternative. Regardless, the EV field can solely benefit from a larger, more homogeneous, cell source to generate EVs, as well as the possibility of generating more EVs per cell, given the realities of delivering cell therapies.

As well as exploring the degree of lipid membrane particles and common EV tetraspanins present in MSC EV enrichments, nano flow cytometry was used to quantify the degree in which common MSC surface markers are incorporated. This revealed that these markers may not be as abundant in EV enrichments, despite confirmation their parental cells have >95% positivity. This is somewhat backed up by a recent review where the only proteomic analysis conducted on UC‐MSCs revealed none of these markers were detected (van Balkom et al. 2019). This could be due to the limited membrane available to incorporate these proteins into EVs, although other sources of MSCs were able to detect these markers in most cases (Théry et al. 2009). Regardless, this could be of concern to those who hypothesised that these markers could readily be used to define, quantify, or establish quality control measures for MSC EVs (Dominici et al. 2006; Witwer et al. 2019). Although this result would certainly need to be confirmed by other MSC EV researchers, it poses the concern that UC‐MSC EVs, at least, may not be as easily identified or distinguished from non MSC EV particulate. It also removes the possibility that CD73 could be used to measure an MSC EV enrichments functionality due to its enzymatic activity. Alternatively, it could present a subset of MSC EVs needing to be further explored. In fact, one study has already concluded CD73 + MSC‐EVs have enhanced immunosuppressive capabilities (Duan et al. 2024). Our findings suggested CD44 was far more abundant, being detected in the EV enrichments of all donor sources using mass spectrometry and could be considered a better candidate for this purpose. It even has established EV associations and therapeutic relevance, having been deemed essential for EV uptake to restore rejected lung transplants and benefit a model of lung injury (Gennai et al. 2015; Hu et al. 2018). However, further research into MSC EV markers would be required to confirm this and would be of great value to the MSC‐EV community.

Further analysis of the MSC EV proteome by mass spectrometry revealed both similarities and differences between single and pooled donor sources. All MSC EV enrichments, regardless of origin, were associated with many terms involved in the formation, transport and uptake of EVs and had the potential to be therapeutically beneficial. This association is supported by other researchers and is the primary rationale behind MSC EV therapy (Chen et al. 2016; Del Fattore et al. 2015; Ma et al. 2019; Zhang et al. 2022). Only the pooled donor source, however, showed an ability to increase the yield of EVs enriched. With transport mechanisms being promoted, it is possible that EVs are working via a positive feedback mechanism to promote their own formation and upregulate communication through their protein cargo. However, without validation of our mass spectrometry findings and the limited proteome detected, further research would be needed to explore this hypothesis. It should also be highlighted that our findings were limited by the lack of a media only or antibody/dye only controls and the presence of bovine proteins due to the use of EV‐depleted FBS, as opposed to a serum free culture. Regardless, each culture would theoretically have the same ‘background’, visualisation suggested samples were majority vesicular, and analysis of human proteins clearly demonstrated their EV nature and highlighted unique pooled source attributes. This included proteins associated with ‘wound healing’ and increased transport mechanisms being detectable in pooled donor samples.

In terms of their unique cargo, pooled EV enrichments showed two significantly upregulated proteins in comparison to single donors, ATP2B1 and basigin. ATP2B1 is an ATP dependant calcium transporter that is crucial for maintaining homeostasis through calcium regulation and nitric oxide signalling, but also promotes osteoblast differentiation (Stafford et al. 2017). Basigin, alternatively known as CD147, is a transmembrane protein that regulates the immune response and matrix metalloproteinase induction but is also commonly found on MSC EVs (Kalra et al. 2012; Pathan et al. 2019; Xiong and L 2014).

Despite these conflicting associations, the delivery of MSC EV enrichments into an inflammatory model of arthritis confirmed their therapeutic benefit. MSC EV enrichments were capable of significantly reducing joint swelling from day 2, which was also reflected in the histopathological outcomes. This suggested that in comparison to their parental cells, MSC EV enrichments are more therapeutically beneficial to an antigen induced model of arthritis. Other studies of autoimmunity have similarly concluded the benefit of delivering MSC EVs to animal models (Cosenza et al. 2018; Fujii et al. 2018), yet those comparing parental cells to EVs showed equivalent benefit (Sharma et al. 2021; Xu et al. 2021).

This observed benefit, mainly reduced joint swelling and histopathological signs, seemed to be amplified when using EV enrichments from a pooled donor source. Since the decision was made to normalise EV enrichments by cell number, this could be due to twice the number of particles being delivered into the joint (due to the observed phenomenon that pooled UC‐MSCs generate more EVs). The possibility that other single donor sources (donors 1, 3 or 4) may have shown better results, despite best efforts to consider MSC characteristics, should also be considered. However, some pooled MSC studies suggest this source could have superior immunomodulatory capabilities (Kuçi et al. 2016). Thus, future experiments should try to overcome these limitations and determine whether these results are an effect of dosage, increased immunomodulatory EV cargo generated upon pooling, or perhaps a combination of the two. Further experimentation would also benefit from larger sample sizes of experimental groups to increase statistical power and confidence in our findings. Functional potency assays, such as T‐cell suppression, macrophage polarisation or cytokine response would also build upon the results which have already been generated to create a wider picture. Regardless, these experiments highlight how donor pooling has not compromised therapeutic efficacy yet offers the benefit of easily generating a larger batch of EVs to treat more mice or offer higher doses.

Throughout, it should also be considered that one pooled donor sample was explored, consisting of a combination of only four single donors, which may not capture full donor heterogeneity. Although pooled donors are thought to be consistent in their properties, even when one donor is removed from the pool (Samuelsson et al. 2009), it is unclear from this study whether the findings presented are solely representative of this ‘batch’, or whether a completely new batch would have consistent characteristics and therapeutic benefit. Therefore, future research should seek to determine whether different combinations of donors will produce similar results and what is the optimal methodology to create donor pools. It would also be enlightening to track individual donors during the pooled culture process to determine if any donor begins to dominate the culture, although the large overlap of protein clusters suggests this is not the case.

## 5 Conclusion

Donor pooling has proven to be a simple and effective method of generating MSC EV enrichments, with a potential to increase MSC EV yields. We have shown donor pooling can encourage an increase in particles with typical EV characteristics, including the presence of known EV markers and common morphology. This could be associated with a positive regulation of protein transport mechanisms, although further work will be needed to confirm this hypothesis. Regardless, this work is valuable to the field of EV research and therapeutics since it provides an easily executed way to scale production through the larger cell source available and increased MSC EV enrichment per cell. It has also begun the extensive work required to understand how donor pooling changes EV enrichment characteristics. Similarly, it provides insights into EV specific paracrine signalling that may occur upon recognition of other donors in culture. More importantly, use of pooled MSCs has not compromised the EV enrichments therapeutic benefit. They were able to effectively reduce joint swelling and histopathological signs of inflammation in an inflammatory model of arthritis. Not only is this knowledge valuable to the future of EV research and therapeutics, but it may also prompt further investigation into the biology behind donor pooling and prove significant to allogeneic MSC therapeutics.

## Author Contributions

**Rebecca Davies**: conceptualization, methodology, data curation, investigation, formal analysis, visualization, writing – original draft, writing – review and editing. **Claire Mennan**: investigation, methodology, supervision, writing – review and editing. **Anais Makos**: investigation, writing – review and editing. **Tian Lan**: investigation, writing – review and editing. **Charlotte Hulme**: formal analysis, supervision, writing – review and editing. **Mark Platt**: methodology, resources, writing – review and editing, supervision. **Karina Wright**: resources, methodology, supervision, writing – review and editing. **Oksana Kehoe**: conceptualization, formal analysis, funding acquisition, methodology, project administration, resources, supervision, writing – review and editing.

## Conflicts of Interest

The authors declare no conflicts of interest.

## Supporting information




**Supporting Information**: jex270167‐sup‐0001‐SuppMat.docx

**Supporting Information**: jex270167‐sup‐0002‐FigureS3.tif

## Data Availability

The data that support the findings of this study are available from the corresponding author upon reasonable request.
