# Supplementary material for: Donor Pooling as an Effective Method to Increase MSC EV Production Without Compromising Therapeutic Potential
Source: J Extracell Biol. 2026 Jul 13;5(7):e70167. doi: 10.1002/jex2.70167 (PMC13364544; doi:10.1002/jex2.70167)
Supplement: Supplementary file 1 — Supporting Information: jex270167‐sup‐0001‐SuppMat.docx [file JEX2-5-e70167-s001.docx]

**Supporting Information**

Supplementary Table 1 – Antibodies used in flow cytometry of UC-MSCs, including an indication to MSC +ve (positive) markers and MSC -ve (negative) markers, and nano flow cytometry of UC-MSC EVs*.*

| **Panel** | **Tube** | **Marker** | **Item Number** | **Dilution** | **Purpose** |
| --- | --- | --- | --- | --- | --- |
| MSC phenotyping | 1 | Unstained | N/A | N/A | Unstained control |
|  | 2 | APC IgG1 | 555751 | 1:100 | Isotype control |
|  |  | PE IgG1 | 555749 | 1:200 | Isotype control |
|  |  | BV421 IgG1 | 562438 | 1:100 | Isotype control |
|  |  | PerCP-Cy5.5 IgG2b | 558304 | 1:100 | Isotype control |
|  | 3 | CD105 | 562408 | 1:100 | MSC +ve marker |
|  |  | CD90 | 555596 | 1:200 | MSC +ve marker |
|  |  | CD73 | 562430 | 1:100 | MSC +ve marker |
|  |  | CD14 | 562692 | 1:100 | MSC -ve marker |
|  | 4 | CD45 | 555483 | 1:20 | MSC -ve marker |
|  |  | CD34 | 555824 | 1:20 | MSC -ve marker |
|  |  | CD19 | 562440 | 1:20 | MSC -ve marker |
|  | 5 | APC IgG2b | 555745 | 1:5 | Isotype control |
|  | 6 | HLA-DR | 559868 | 1:5 | MSC -ve and  immunogenic marker |
|  | | | | | |
| MSC immuno-phenotyping | 7 | PE IgG1 | 555749 | 1:20 | Isotype control |
|  | 8 | CD40 | 560963 | 1:20 | Immunogenic marker |
|  | 9 | CD80 | 560925 | 1:50 | Immunogenic marker |
|  | 10 | CD86 | 560957 | 1:50 | Immunogenic marker |
|  | 11 | CD106 | 561679 | 1:50 | Immunomodulatory marker |
|  | | | | | |
| MSC EV phenotyping | N/A | MemGlow™ | NG01-10 | 1:500 | Plasma membrane dye |
|  | N/A | CD9  CD63  CD81 | ab18241 ab18235  ab239256 | 1:300 | EV markers |
|  |  | | | | |
|  | N/A | CD73 | ab155378 | 1:50 | MSC +ve marker |
|  | N/A | CD90 | ab139364 | 1:50 | MSC +ve marker |
|  | N/A | CD105 | ab155367 | 1:50 | MSC +ve marker |
|  | N/A | HLA-DR | ab239283 | 1:50 | MSC -ve and  immunogenic marker |


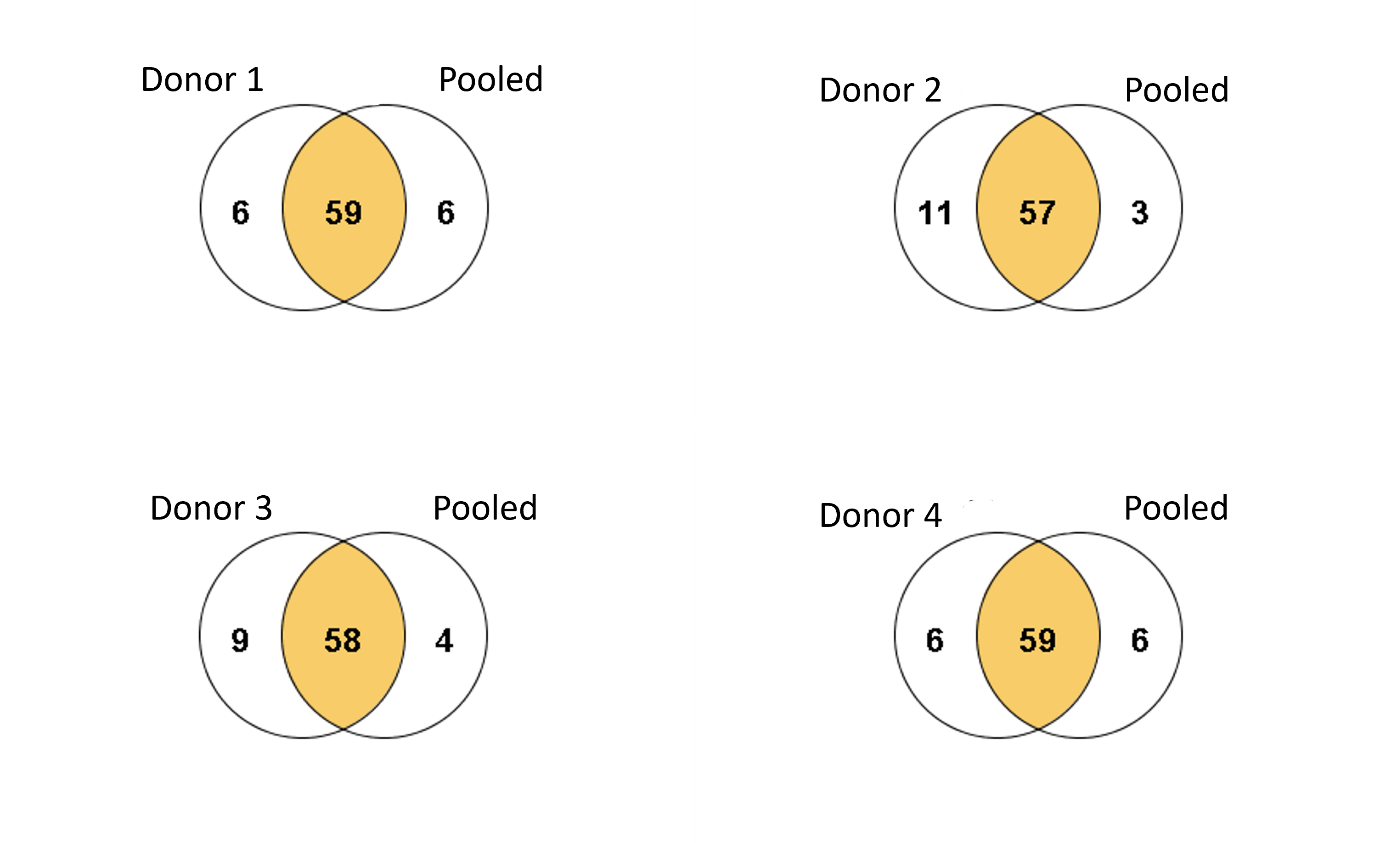


Supplementary Figure 1 – Protein cluster analysis of single donors compared to the pooled donor source, showing the number of protein clusters which are not significantly different (orange) and those that are significant (white, p<0.05).


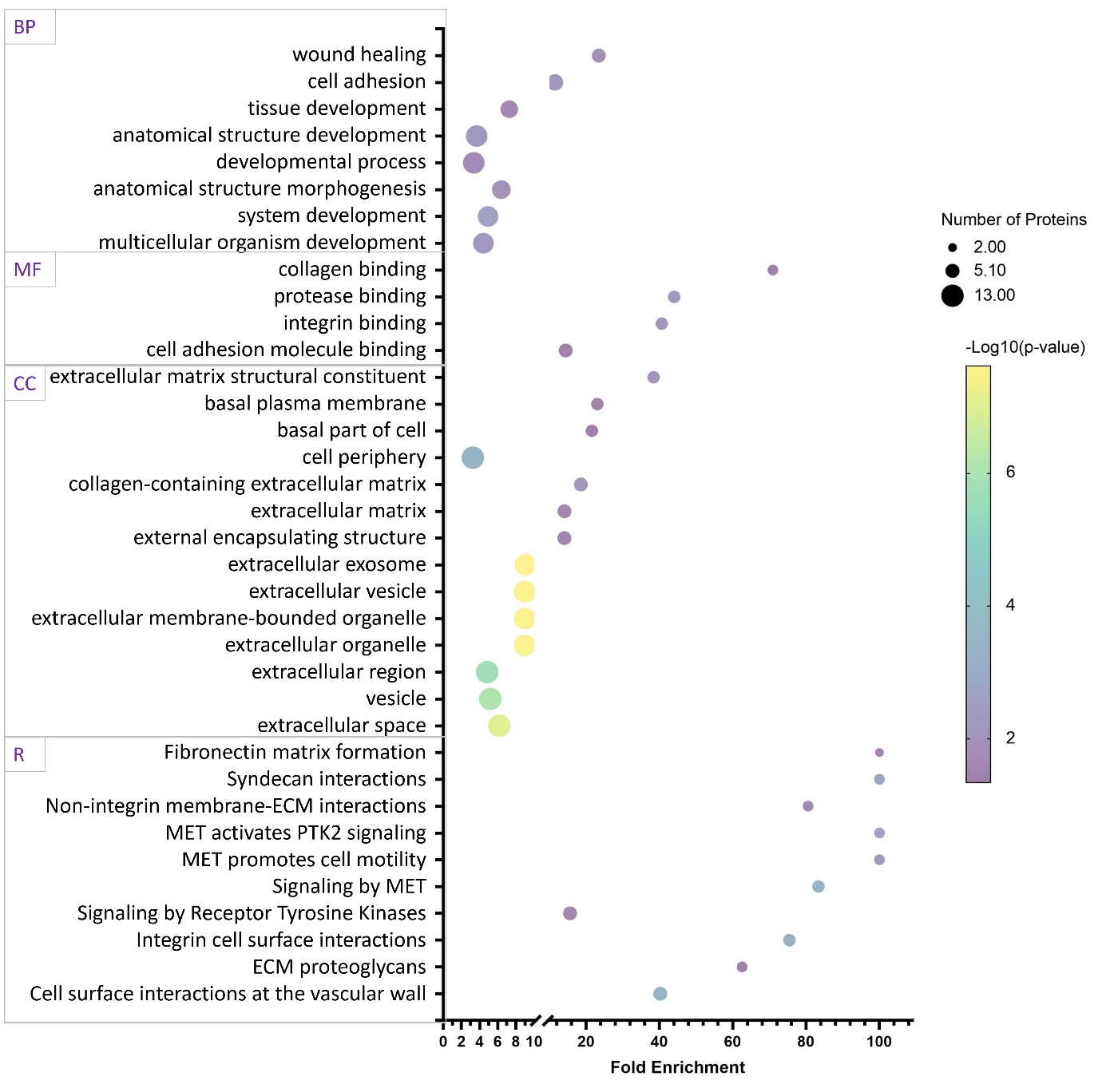
Supplementary Figure 2 - Significantly enriched terms identified in GO analysis of common proteins found in all UC-MSC EV samples*.* This is regardless of source, thereby including all four single donor, and pooled donor, UC-MSC EV samples. Biological process (BP) cellular component (CC), molecular function (MF), reactome pathway (R) terms associated with the human proteins identified in MSC EV proteomic analysis are displayed on the y-axis, whilst the x-axis displays the fold enrichment of that term in comparison to the complete human proteome. The significance of this enrichment can be determined by the colour of the dot, which has been log transformed, and size correlates with the increasing number of proteins associated with each term found in this analysis.
