# Supplementary figures and images for: Donor Pooling as an Effective Method to Increase MSC EV Production Without Compromising Therapeutic Potential
Source: J Extracell Biol. 2026 Jul 13;5(7):e70167. doi: 10.1002/jex2.70167 (PMC13364544; doi:10.1002/jex2.70167)

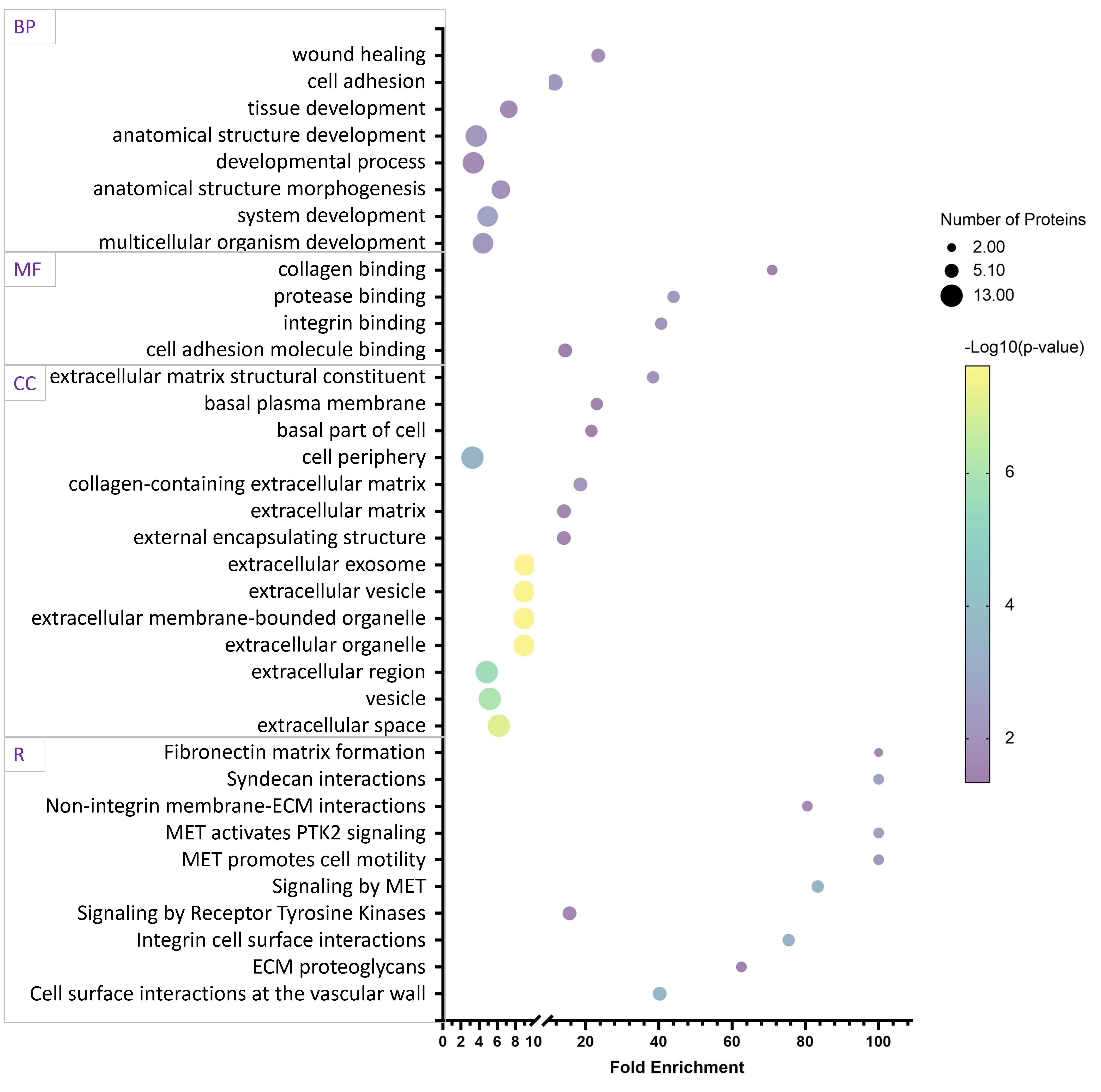

Supplement: Supplementary file 2 — Supporting Information: jex270167‐sup‐0002‐FigureS3.tif [file JEX2-5-e70167-s002.tif]
